# Teaching games for understanding in school handball: a controlled pre-post study with intact Brazilian physical education classes

**DOI:** 10.3389/fpsyg.2026.1817709

**Published:** 2026-05-19

**Authors:** Rodrigo Márcio de Oliveira Silva, Lucas Savassi Figueiredo, Filipe Manuel Clemente, Adam Kawczyński, Cláudio Farias, José Carlos Pontes Corrêa, Eduardo de Paula Amorim Borges, Gustavo Ferreira Pedrosa, Juracy da Silva Guimarães, Lorenzo Laporta, Gustavo De Conti Teixeira Costa

**Affiliations:** 1 Núcleo de Estudo e Pesquisa Avançada em Esportes, Faculdade de Educação Física, Universidade Federal de Goiás, Goiânia, Brazil; 2 Centro de Estudos de Cognição e Ação, Escola de Educação Física, Fisioterapia e Terapia Ocupacional, Universidade Federal de Minas Gerais, Belo Horizonte, Brazil; 3 Gdansk University of Physical Education and Sport, Gdańsk, Poland; 4 Sport Physical Activity and Health Research and Innovation Center, Coimbra, Portugal; 5 Applied Research Institute (I2A), Polytechnic University of Coimbra, Coimbra, Portugal; 6 Faculty of Medicine, Wrocław University of Science and Technology, Wrocław, Poland; 7 Faculdade de Desporto, Universidade do Porto, Porto, Portugal; 8 Núcleo de Estudos em Performance Analysis Esportiva, Centro de Educação Física e Desportos, Universidade Federal de Santa Maria, Santa Maria, Brazil

**Keywords:** decision-making, executive functions, game-centered pedagogy, intrinsic motivation, self-determination theory, sport psychology

## Abstract

**Introduction:**

This controlled pre-post study with intact school classes examined whether participation in a TGfU-based handball unit was associated with motivational, cognitive, and technical-tactical changes compared with Direct Instruction and a comparison condition in Brazilian public-school physical education.

**Methods:**

Sixty-three students aged 11–13 years from three intact classes were allocated to TGfU handball, Direct Instruction handball, or a comparison volleyball unit, with one class per condition. The intervention comprised 20 lessons of 50 min. Outcomes were assessed before and after the instructional unit and included motivation for physical activity participation (MPAM-R), cognitive flexibility assessed exploratorily using the Stroop test, and technical-tactical indicators observed during small-sided handball games. Data were analyzed using nonparametric longitudinal models based on Wald-Type Statistics, with *post-hoc* comparisons and effect sizes.

**Results:**

Enjoyment showed a Group × Time effect, WTS(2) = 85.60, *p <* 0.001, increasing only in the TGfU group (4.69 ± 0.81 to 6.42 ± 0.46; *p <* 0.001, r = 0.876). Competence also showed a Group × Time effect, WTS(2) = 28.02, *p <* 0.001, increasing only in the TGfU group (4.77 ± 1.06 to 5.99 ± 1.08; *p <* 0.001, r = 0.846). Stroop outcomes improved over time, but no Group × Time effects were observed. For technical-tactical outcomes, the TGfU group reduced inaccurate passes (1.71 ± 1.38 to 0.43 ± 0.75; *p* = 0.002, r = 0.667) and increased occupying open spaces (1.19 ± 1.63 to 3.86 ± 3.65; *p* = 0.001, r = 0.698), whereas the Direct Instruction group decreased accurate passes (20.76 ± 8.90 to 10.14 ± 5.05; *p <* 0.001, r = 0.831).

**Discussion:**

A TGfU-based handball unit was associated with favorable changes in intrinsic motivation-related dimensions and selected offensive tactical behaviors in this school context. No condition-specific changes in cognitive flexibility were observed, suggesting that Stroop performance changes were not specific to TGfU. Because allocation occurred at the class level with one class per condition and without blinding, the findings should be interpreted as contextual associations rather than causal estimates.

## 1 Introduction

Motivation and executive functions are two psychological constructs central to adolescent development and long-term engagement in physical activity (Deci and Ryan, 2000; Diamond, 2013). Adolescence is a particularly sensitive period during which motivational orientations, whether self-determined or externally regulated, consolidate and exert lasting influence on health behaviors, academic persistence, and sport participation (Deci and Ryan, 2000). Concurrently, executive functions such as cognitive flexibility and inhibitory control undergo substantial maturation during this period, with implications for academic achievement, social functioning, and the capacity to navigate complex, unpredictable environments (Diamond, 2013). Physical education (PE) is uniquely positioned to address both of these developmental priorities: as the only school curriculum subject mandated to promote physical and motor development, it also holds distinct potential for cultivating students’ psychological wellbeing, intrinsic motivation, and cognitive growth through structured movement and game-based learning (Chen et al., 2020; Mitchell and Walton-Fisette, 2022). Yet this potential is rarely realized. Large-scale and cross-national analyses consistently indicate that PE as typically enacted in schools has not provided the pedagogical conditions required to support positive motivational climates, self-determined engagement, or broader cognitive outcomes (Aelterman et al., 2019). Regular PE is frequently characterized by limited instructional intentionality, inconsistent task design, and reduced curricular time, conditions that tend to undermine rather than foster students’ intrinsic motivation and psychological engagement in learning (Hardman and Green, 2011; Casey, 2022). Against this backdrop, the choice of a pedagogical model becomes a critical determinant of whether PE fulfills its psychological and developmental promise.

When instructional structure is sought through pedagogical models, Direct Instruction, primarily based on behaviorist-oriented, analytical, and directive instruction, remains the dominant approach in games and sport-related teaching (Donoghue and Hattie, 2021). In Direct instruction, learning to play games progresses through bottom-up, molecular approaches to content development, in which discrete motor abilities (e.g., shooting, passing, dribbling, supporting) are taught in isolation. Gameplay is typically placed at the end of the lesson as a culminating activity, where students are expected to integrate and apply previously learned skills in response to the game’s demands. Heavily relying on direct instruction and pre-established content and gameplay solutions, students are largely tasked with rote repetition of teacher-defined content, in which the teacher assumes control over all key elements of the teaching–learning process, including content delivery, feedback, and pacing (Metzler and Colquitt, 2021). Although Direct Instruction may facilitate gains in game performance related to technical execution, at the early stages of learning a sport, evidence indicates limitations in transferring these skills to authentic game situations, particularly in decision-making, tactical understanding, and student engagement (Stolz and Pill, 2014). Accordingly, its impact appears primarily motor in nature, with little evidence of systematic effects on cognitive, social, psychological, or motivational domains.

In contrast, Teaching Games for Understanding (TGfU), in which students learn the game through gameplay, has increasingly established itself as one of the most influential game-based approaches in sport pedagogy and PE (Kirk, 2017; Morales-Belando et al., 2022). TGfU frames learning as a process of developing students’ ability to resolve emergent gameplay problems in reference to games’ core tactical principles (e.g., creating, using, and defending space). Through the design of developmentally appropriate learning tasks representative of the mature version of sports, such as modified and small-sided games, and the systematic use of tactical questioning, students are led to develop a deep appreciation and understanding of the game, as well as the ability to select (tactical decision making) and enact (technical skill execution) the most appropriate technical and tactical responses across diverse game situations (Almond, 2015; Farias et al., 2015; Pan et al., 2023). This approach is expected to simultaneously favor game reading (perception), decision making (what, when, where to do) and skill execution (how to do) (Ginciene et al., 2023; Ribas et al., 2023).

Decision-making and skill execution represent core technical-tactical variables through which the differential effects of pedagogical models in games teaching have been examined. Decision-making in sport contexts refers to the ability to select the most appropriate action among available options in response to the configuration of the game at a given moment, integrating perceptual recognition, tactical knowledge, and anticipatory reasoning (Raab, 2003). Skill execution, in turn, refers to the motor enactment of the selected action with adequate efficiency and spatial–temporal accuracy (French and Thomas, 1987). TGfU has been theorized to favor decision-making development by situating learners within authentic game problems that demand continuous perceptual monitoring, tactical reasoning, and adaptive responding (Kirk and MacPhail, 2002; Farias et al., 2015). Research on TGfU has consistently shown improvements in tactical knowledge, decision-making, and overall game performance across different invasion sports (González-Valero et al., 2024; Morales-Belando et al., 2022; Pan et al., 2023). By contrast, Direct Instruction’s sequential, skill-first progression is expected to support technical execution in isolated conditions but may offer fewer opportunities to develop the contextual cue recognition and rapid decision processes required in dynamic game situations (Stolz and Pill, 2014). Empirical evidence broadly supports these expectations: studies comparing TGfU and Direct Instruction have generally reported advantages for TGfU in decision-making measures, while findings for skill execution are more mixed, with some studies favoring Direct Instruction at early learning stages and others showing comparable outcomes across models (Harvey et al., 2020; González-Valero et al., 2024). These patterns suggest that the relative pedagogical value of each model may depend on the specific outcome prioritized, the learners’ developmental stage, and the duration of the instructional unit, highlighting the importance of assessing both decision-making and execution concurrently within the same study design.

The technical-tactical outcomes described above are not independent of the motivational climate in which learning occurs. The degree to which students engage persistently with game tasks, invest cognitive effort in tactical problem-solving, and seek to refine their skill execution is substantially shaped by their motivational orientation toward the activity (Deci and Ryan, 2000). From a Self-Determination Theory perspective (Deci and Ryan, 2000), motivational quality in PE is determined by the degree to which the social and instructional environment satisfies students’ basic psychological needs for autonomy, competence, and relatedness. TGfU’s pedagogical architecture centered on appropriately modified games, tactical questioning, and student-led problem-solving is theoretically aligned with need-supportive teaching, as it fosters perceived autonomy through choice and self-regulation, promotes competence through graduated challenge and visible tactical progress, and enhances relatedness through cooperative and competitive game contexts (González-Valero et al., 2024; Saiz-González et al., 2024; Flores-Cidoncha et al., 2025). Empirical evidence broadly supports this alignment: studies conducted in secondary school PE have reported that TGfU conditions are associated with higher levels of intrinsic motivation, identified regulation, enjoyment, and effort compared to technique-first approaches (Morales-Belando et al., 2022; Saiz-González et al., 2024). Conversely, Direct Instruction, by emphasizing external regulation, teacher-controlled pacing, and prescriptive feedback, has been associated with more controlled motivational profiles and lower perceived autonomy, particularly among adolescent learners (Aelterman et al., 2019; Haerens et al., 2018). Nonetheless, some studies have reported null or mixed motivational effects between models, particularly in short-term interventions or contexts where students lack prior familiarity with game-based formats, underscoring the need for ecologically sensitive comparisons (Breed et al., 2025).

Beyond motivational factors, executive functions, and particularly cognitive flexibility, have attracted increasing attention as potential outcomes of structured physical activity programs in school contexts (Diamond, 2013; Harvey et al., 2020). Cognitive flexibility refers to the capacity to shift attentional focus, adapt behavioral responses, and reorganize cognitive strategies in the face of changing task demands. This particular function is considered essential for performance in open, unpredictable sport environments (Krenn et al., 2018; Brimmell et al., 2024). Theoretically, TGfU’s emphasis on dynamic, decision-rich game situations is expected to generate the kind of sustained cognitive engagement (involving rapid perception, rule-based problem solving, and adaptive responding) that may stimulate executive control systems (Harvey et al., 2020; Nopembri et al., 2022). This proposition finds partial support in research on “cognitively engaging physical activity” programs, which have reported improvements in executive functions in school settings (Vazou et al., 2016; Kolovelonis et al., 2022).

However, a substantive methodological challenge in this area concerns the ecological validity of the instruments employed to assess cognitive flexibility. Standardized neuropsychological tasks (e.g., Stroop test) measure executive control in decontextualized, laboratory-like conditions that may not capture the sport-specific cognitive demands experienced during gameplay (Moreau and Conway, 2014). This may partly explain why the existing evidence base linking game-based pedagogical models specifically to cognitive flexibility remains limited and inconsistent. While the construct may be relevant, the instruments conventionally available may be insufficiently sensitive to the contextual nature of executive demands in sport. Acknowledging this tension, the present study employs a standardized measure of cognitive flexibility as a first-level indicator of potential domain-general transfer effects, while recognizing that more ecologically grounded assessments represent an important direction for future research.

While recent systematic reviews have broadly reinforced the educational benefits of TGfU, they have also pointed to several methodological and reporting limitations in the existing body of research, including heterogeneity in intervention protocols, variability in implementation fidelity, and limited detail regarding the pedagogical content actually developed during lessons (Ortiz et al., 2023; Breed et al., 2025). A critical methodological consideration concerns the operationalization of technical-tactical outcomes. The GPAI (Oslin et al., 1998) is widely used and has been refined over decades. However, concerns have been raised regarding the inferential ambiguity of its “decision-making” component, since appropriate decisions as coded by observers cannot be unambiguously distinguished from situationally adequate responding (Memmert and Harvey, 2008). In addition, GPAI coding has known limitations in inter-rater reliability in the absence of extensive observer training, and it does not readily yield fine-grained, sport-specific frequency data on individual technical-tactical actions. Thus, in the present study, we adopted a notational analysis protocol based on IHF scouting standards (Schorer et al., 2020; Ferrari et al., 2020), which allows for direct quantification of specific actions (passes, receptions, off-ball movements) in a standardized game format. By foregrounding sport-specific, observable behavioral indicators with discriminant validity (Krawczyk et al., 2025), this approach prioritizes ecological validity and replicability in the context of handball PE units, while acknowledging that the GPAI remains a valuable instrument for broader, cross-sport applications.

The Brazilian context presents a particularly relevant and underexplored setting for TGfU research. Despite the country’s long-standing tradition in team sports and its national curriculum framework that explicitly advocates for active, student-centered, and culturally meaningful pedagogies in PE (Brazil Ministry of Education, 2018), the adoption of game-based models such as TGfU in Brazilian schools remains limited, particularly in teaching modalities less familiar to students, such as handball (Mazzardo et al., 2020; Mazzardo et al., 2022). Public schools in Brazil, particularly in rural areas, typically struggle with outdated facilities, limited access to sport-specific equipment and restricted curricular time (typically two 50-min sessions per week). Due to resource limitations, Brazilian public school PE teachers usually face challenges in implementing a comprehensive curriculum, affecting the quality and extent of its delivery. Historically, Brazilian PE has been shaped by a sport-technical tradition that prioritizes skill execution and sport performance, often at the expense of student-centered approaches and tactical understanding (Gonzáles and Fensterseifer, 2009). In most Brazilian public schools, teachers receive limited pre-service training in game-based approaches and may encounter institutional barriers to shifting from technique-focused content to more holistic pedagogies. As a result, TGfU is often unfamiliar not only to students but also to the teachers who would implement it. Moreover, implementing TGfU with fidelity in Brazilian public schools requires adapting its core principles to resource-constrained and institutionally conservative environments, a challenge that makes intervention design, implementation monitoring, and context-sensitive reporting especially important. Given these challenges, this study aims to explore how game-based models, particularly TGfU, can be effectively integrated into the Brazilian physical education context, seeking to contribute valuable insights into the effectiveness of TGfU in fostering a more dynamic, student-centered approach to physical education in Brazil.

Considering this background, the present study aimed to analyze and compare the associations of a handball instructional unit based on TGfU, Direct Instruction, and a comparison group that received volleyball lessons with motivational dimensions, exploratory cognitive outcomes assessed through the Stroop test, and technical-tactical indicators observed in a small-sided game context among middle-school students from a Brazilian public school. We hypothesized that the TGfU condition would be associated with greater improvements in motivational outcomes and selected tactical behaviors than Direct Instruction and the comparison condition. We also expected both handball groups to show greater improvements than the comparison group in selected technical-tactical indicators. Cognitive flexibility was examined exploratorily as a potential domain-general transfer outcome, given the Stroop test’s limited ecological specificity for capturing game-based cognitive demands.

## 2 Methods

### 2.1 Sample size and attendance

Participants were students aged 11–13 years enrolled at a public school in Monte Carmelo, Minas Gerais, Brazil. Eligibility criteria for students were: (i) regular attendance in PE classes, (ii) no previous structured experience in handball instruction, and (iii) absence of medical restrictions preventing participation. No students were excluded. A total of 63 students were included in the final analyses: TGfU (*n =* 21), Direct Instruction (*n =* 21), and Comparison (*n =* 21). Participants reported no prior systematic training experience in handball or other collective sports, whether at the school level or in federated clubs. This reduces, but does not eliminate, the possible influence of previous informal experiences with other invasion games or team-sport activities. Because tactical principles such as creating space, supporting the ball carrier, and defending passing lines may transfer across invasion sports, prior informal exposure cannot be fully ruled out as a contributor to the technical-tactical outcomes. While no *a priori* sample size calculation was performed due to the authentic school context, a *post-hoc* power analysis was conducted to assess the adequacy of the sample size. The results showed that the sample size was sufficient to detect significant effects for most outcomes, with power estimates generally exceeding 0.80. However, specifically for offensive and defensive tactical actions, the power was closer to 0.6, suggesting a moderate ability to detect small effects. While these results still provide valuable insights, the lower power in certain comparisons should be considered when interpreting the findings. To enhance interpretability given this pragmatic sample, results are reported with an emphasis on the magnitude and direction of changes across outcomes rather than statistical significance alone. Attendance was high: all students attended at least 18 of the 20 PE lessons delivered during the instructional unit.

### 2.2 Study design and procedures

This study adopted a pre-post quasi-experimental design with intact school classes (clusters) as the unit of allocation across three arms. The design followed a pragmatic school-based approach, preserving the natural organization of classes within the physical education curriculum. Eligibility criteria for classes were as follows: (i) 7th-grade classes, (ii) no prior exposure to handball within the school curriculum, (iii) no previous experience with the TGfU model, (iv) classes taught by the same physical education teacher, and (v) two scheduled physical education sessions per week. Based on these criteria, three existing classes were allocated at the class level to one of three conditions: (i) TGfU (handball taught using the Teaching Games for Understanding approach; *n =* 21), (ii) Direct Instruction (handball taught using a Direct Instruction model; *n =* 21), and (iii) Comparison (volleyball delivered as part of regular PE classes; *n =* 21). The comparison group followed the school’s regular PE curriculum, which comprised a volleyball unit during the study period. Class allocation to the three study arms was performed prior to the beginning of the instructional unit using a simple random draw with paper slips placed in sealed opaque envelopes (one envelope per study arm). The draw and assignment were performed by the first author. Because only one class was allocated to each condition, cluster-level variance could not be estimated separately from the instructional condition. Due to the nature of the pedagogical interventions, participants and the teacher could not be blinded to group allocation. The intervention comprised 20 physical education lessons, each lasting 50 min, conducted on a 28 × 15 m court, and delivered as part of the regular curriculum.

All three study arms were taught by the same PE teacher, thereby reducing potential teacher-related confounding. This professional holds a degree in Physical Education and has 17 years of experience working as a teacher in the school environment. Prior to the study, he had received training in undergraduate and graduate courses focused on the application of the teaching models used in this research, ensuring his qualifications to implement the various programs effectively.

For this study, a didactic unit was created for each group, defined as a set of teaching objectives centered on a core idea. This unit was constructed on the basis of Bayer’s (1994) theory of operational principles in team sports. The objectives of these units for the TGfU were to develop offensive skills related to maintaining possession, advancing toward the goal, and scoring, as well as defensive skills such as recovering possession, preventing or delaying the opponent’s progression, and protecting the goal. For the DI groups, analogous objectives and content were elaborated based on the sport modality and training characteristics. The full 20-lesson didactic unit for the TGfU handball, Direct Instruction handball, and Direct Instruction volleyball conditions is provided in Supplementary material 1. This Supplementary material 1 details, lesson by lesson, the broad pedagogical objectives, specific content, tactical principles, guiding questions, and game formats used in each condition, allowing readers to map the intervention description in the main text onto the actual lesson structure.

**Figure 1:**
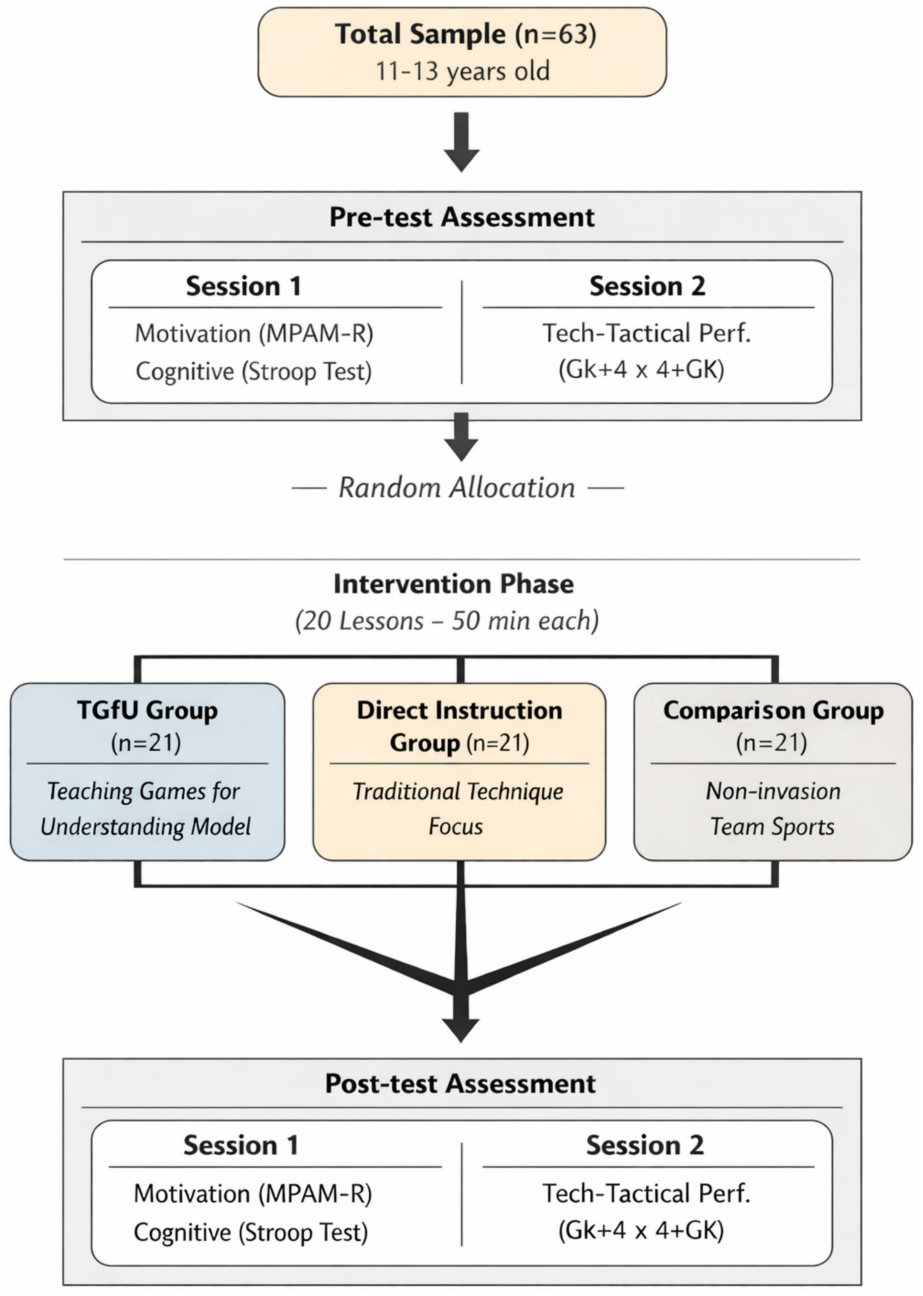
Study design and assessment timeline.

Assessments were conducted before (pre-test) and after (post-test) the instructional unit across two consecutive days. On day 1, students completed the motivation questionnaire and performed the cognitive flexibility test. On day 2, students participated in a goalkeeper + 4 vs. 4 + goalkeeper (G + 4 vs. 4 + G) game on a 28 × 15 m court following the official rules for 15 min. For the game assessments, teams were randomly formed while ensuring two students of each sex per team; goalkeepers were female students and remained the same throughout the games. Post-tests followed the same sequence as pre-tests, maintaining the same team composition (Figure 1). Prior to study initiation, parents/guardians provided written informed consent, and students provided written assent. The study was approved by the Research Ethics Committee of the Federal University of Goiás (protocol no. 2,394,440).

### 2.3 Lesson categorization

To verify the instructional model implemented in PE lessons across the three groups, lessons were categorized using an adaptation of Stefanello (2000) activity categorization protocol. This observational protocol allows the analysis of parameters such as: (i) training segment, (ii) activity identification, (iii) duration, (iv) space delimitation, (v) tasks, (vi) task conditions, (vii) coach/teacher behavior, (viii) player behavior, (ix) drills, (x) success criteria, among other aspects.

All 20 lessons comprising the instructional unit were video-recorded using a camera installed 4 m above the ground at a diagonal angle, providing full visibility of the lesson area. To verify instructional fidelity, lessons were monitored using video recordings and structured observational coding. Two trained raters, both researchers with degrees in Physical Education and over 5 years of experience in school physical education, independently coded the videos by classifying each lesson segment according to predefined task categories (e.g., modified games, isolated drills, questioning episodes, and full games). Segment durations were recorded in minutes, summed within each category, and converted into percentages of total lesson time. Inter-rater agreement was high (Cohen’s kappa > 0.90).

### 2.4 Intervention

#### 2.4.1 TGfU condition

For the TGfU group, the didactic unit emphasized situated activities, such as small-sided games and decision-making tasks with lower complexity. These activities were designed to simulate game situations where players could engage in tactical decision-making and, when necessary, technical skills. The phases of the revisited TGfU model (Kirk and MacPhail, 2002) were applied throughout the lessons, including game form, game concept, thinking strategically, decision-making, movement execution, and situated performance. While the movement execution phase was only implemented in a few lessons, the other phases were present in all sessions. The TGfU core principles of sampling, exaggeration modifications, game representation, and gradual increases in complexity were also applied throughout the lessons, ensuring a progressive and contextualized learning experience (Holt et al., 2002).

All lessons were video-recorded, segmented, and coded into predefined task categories; segment durations were summed and expressed as a percentage of total lesson time. In the TGfU unit, lesson activities were organized around tactical problems and delivered predominantly through tactical-technical activities (36.00%) and small-sided/modified games (37.72%). The remaining 26.28% of lesson time (i.e., 100–36.00 - 37.72) corresponded to other coded lesson segments (e.g., warm-up/organization and transitions/management), ensuring that the coded distribution sums to 100%.

#### 2.4.2 Direct instruction condition

Direct Instruction followed a technique-first progression, centered on teacher demonstration and isolated technical drills (e.g., passing, catching, dribbling, shooting) with prescriptive feedback, progressing from low- to higher-complexity drill sequences and only later to more game-related activities (Metzler and Colquitt, 2021). Fidelity coding indicated that isolated technical drills represented 53.33% of total lesson time. The remaining 46.67% (i.e., 100–53.33) comprised other coded lesson segments (e.g., warm-up/organization, transitions/management, and game-related segments when implemented), so that the distribution sums to 100% by design.

#### 2.4.3 Comparison condition

The comparison group completed a regular PE unit in volleyball, delivered under a traditional, technique-focused model, similarly to the Direct Instruction condition (Metzler and Colquitt, 2021). To ensure comparability with the Direct Instruction unit, the lesson structure was designed to mirror its time allocation: approximately 53.33% of lesson time was dedicated to isolated technical drills (e.g., overhead pass and forearm pass) and the remaining 46.67% allocated to the other lesson segments such as warm-up/organization, transitions/management, and game-play tasks (e. g., 6v6). This allocation totaled 100% of the lesson time.

### 2.5 Instruments

#### 2.5.1 Motivation for sport participation

Motivation for sport participation was assessed using the Motivation for Physical Activities Measure-Revised (MPAM-R; Ryan et al., 1997), in its Brazilian Portuguese version adapted and validated by Albuquerque et al. (2017). The MPAM-R is a self-report scale designed to assess the strength of five motivational dimensions for physical activity participation. Items are rated on a 7-point Likert scale anchored at 1 (‘not at all true for me’) and 7 (‘very true for me’). Subscale scores are computed as the arithmetic mean of the constituent items, yielding a score between 1 and 7 for each dimension, with higher scores reflecting stronger endorsement of the corresponding motive.

In the Brazilian Portuguese version of the MPAM-R the five subscales are: (1) Enjoyment (7 items; e.g., “Because I enjoy this activity”), which captures the intrinsic motive of engaging in physical activity because it is fun, interesting, and pleasurable; (2) Competence (4 items; e.g., “Because I want to improve at my activity”), reflecting the desire to improve one’s skills, meet challenges, and develop mastery; (3) Fitness (4 items; e.g., “Because I want to be physically healthy”), capturing health and physical condition as motives; (4) Appearance (6 items; e.g., “Because I want to improve my body shape”), reflecting externally oriented motives related to physical appearance; and (5) Social (5 items, e.g., “Because I want to meet new people”), reflecting participation for social interaction and affiliation. Of the five subscales, Enjoyment and Competence reflect intrinsic motivational orientation, whereas the other three subscales (Fitness, Appearance, and Social) reflect levels of extrinsic motivation.

#### 2.5.2 Cognitive flexibility

Cognitive flexibility was assessed using the Color and Word Stroop Test developed by John Ridley Stroop (Stroop, 1935). The Stroop Color and Word Test can be used as a measure of cognitive flexibility (Golden, 1978). The protocol first included “Word Reading,” in which students read 112 words (color names) printed in black; the number of correct responses (words read accurately) and the time required to complete the task were recorded (time limit: 120 s). Subsequently, students performed “Color Naming,” in which they named the ink color of 112 color words presented in incongruent colors; the number of correct responses (colors named accurately) and the time required to complete the task were recorded (time limit: 120 s).

#### 2.5.3 Technical-tactical behavior analysis

Technical-tactical behavior was assessed via notational analysis, consistent with previous investigations (Schorer et al., 2020). Games were analyzed in a G + 4v4 + G format on a 28 × 15 m court. Each game lasted 15 min, and teams were instructed to adopt man-to-man defense in the defensive half. The frequency of each study variable was recorded. To assess technical fundamentals, scouting models adapted from the International Handball Federation models were used. Accordingly, the following technical elements and indicators were analyzed.

For the evaluation of game fundamentals, occurrences of passes, receptions, and shots within the attacking half were recorded as follows:

*Pass effectiveness*: passes were classified as accurate when the ball was delivered in a controlled manner to a teammate, and inaccurate when the ball did not reach a teammate.

*Reception effectiveness*: a reception was classified as successful when the player maintained possession, allowing continuity of play, and unsuccessful when ball control was not maintained, preventing continuation of the action.

*Shot effectiveness*: outcomes were classified as a goal when the ball fully crossed the goal line, or an off-target shot when the attempt did not hit the goal.

#### 2.5.4 Offensive and defensive tactical actions

For offensive tactical actions performed by players with and without ball possession, the frequency of the following individual tactical actions within the attacking half was recorded:

Off-ball movements to create passing options: when the player moved to receive the ball, enabling a precise pass from a teammate with minimal risk of ball loss (Gryko et al., 2020);

Movements after passing: when the player moved after passing the ball, positioning themselves favorably to receive the ball again (Ferreira et al., 2018);

Occupying open spaces: when the player moved into an open space that allowed them to receive the ball and continue the attack (Farias et al., 2018);

Shots on goal: actions in which the player directed the ball effectively toward the opponent’s goal (Aquino et al., 2018).

For defensive tactical actions, the frequency of the following variables within the defensive half was recorded:

Pressuring the ball carrier: when the defender moved to control/pressure the attacker in possession of the ball (Ferreira et al., 2018);

Tracking an off-ball opponent: when the defender moved to mark an attacker without the ball who was moving toward an area/position favorable for receiving the ball (Ferreira et al., 2018);

*Interceptions*: when the defender anticipated the pass trajectory, preventing the ball from reaching the intended target (Aquino et al., 2018);

*Steals*: when the defender, in a dribbling situation, applied close marking and recovered the ball from the opponent (Daza et al., 2017);

*Shot blocking*: when a defender blocked the ball’s trajectory after a shot attempt (Daza et al., 2017).

### 2.6 Reliability of technical-tactical behavior data

All games were recorded from an elevated overhead perspective using a GoPro 8 Black (GoPro, Inc.) at 1080p HD resolution and a 60 Hz sampling rate. The camera was positioned approximately 4 m above the ground at a diagonal angle, capturing the entire playing area. Two PE professionals with more than 5 years of experience in handball performed the coding of the actions assessed in the study. For reliability testing, 30% of the actions were reanalyzed, exceeding the 10% reference value (Tabachnick and Fidell, 2013). Intra-observer Cohen’s kappa values were >0.90 with standard errors <0.05. Inter-observer analysis yielded values >0.95 with standard errors <0.04. These values exceed the recommended threshold of 0.75.

### 2.7 Statistical analysis

Normality of the variables was assessed using Shapiro–Wilk tests and visual inspection, indicating systematic violations of parametric assumptions. Considering the repeated-measures structure (PRE vs. POST within the same participants), the presence of three independent groups (TGfU, Direct Instruction, Comparison), and the distributional violations described above, the most appropriate inferential approach was a nonparametric factorial repeated-measures design. Accordingly, group comparisons over time were conducted using the Wald-Type Statistic (WTS) implemented in the nparLD package in R (v4.3), which is suitable for nonparametric longitudinal data and does not rely on assumptions of normality or sphericity (Noguchi et al., 2012). Given the nonparametric nature of the data and the use of WTS, effect sizes were reported only for *post-hoc* comparisons. This approach was adopted because standardized effect size measures for global nonparametric longitudinal tests remain limited and lack straightforward interpretability. When a significant main effect of Group was identified, *post-hoc* comparisons were performed using Dunn’s test with Holm correction for multiple comparisons to locate between-group differences. When the Group × Time interaction was significant, paired Wilcoxon tests were used as complementary *post-hoc* analyses, as they are appropriate for within-group repeated-measures comparisons. In these cases, r values were reported as effect-size estimates and interpreted as small (≈0.10), medium (≈0.30), and large (≈0.50) (Fritz et al., 2012). Statistical significance was set at *α* = 0.05 for all analyses. Data preparation and effect-size calculations were performed in Python (v3.10), and inferential analyses were conducted in R (v4.3). Given the single-cluster-per-arm design, results are interpreted primarily in terms of the magnitude and consistency of changes across outcomes rather than as definitive causal estimates.

## 3 Results

### 3.1 Motivation for sport participation

Results for motivation toward sport participation/PE lessons (Table 1) are presented according to the MPAM-R dimensions. Analysis of the Enjoyment dimension indicated a significant effect of Time, WTS(1) = 11.32, *p* = 0.0008, but not of Group, WTS(2) = 3.63, *p* = 0.163. A significant Group × Time interaction was also identified, WTS(2) = 85.60, *p <* 0.001. The TGfU group showed an increase in this dimension (*p <* 0.001, r = 0.876), whereas the Direct Instruction (*p* = 0.244, r = 0.254) and Comparison (*p* = 0.375, r = 0.193) groups showed no changes. For the Competence dimension, the analysis indicated a significant effect of Time, WTS(1) = 8.31, *p* = 0.004, but not of Group, WTS(2) = 1.12, *p* = 0.572. A significant Group × Time interaction was also identified, WTS(2) = 28.02, *p <* 0.001. An increase was observed in the TGfU group (*p <* 0.001, r = 0.846), whereas the Direct Instruction (*p* = 0.566, r = 0.125) and Comparison (*p* = 0.330, r = 0.212) groups showed no changes. For the Appearance dimension, the analysis indicated a significant effect of Time, WTS(1) = 4.15, *p* = 0.042, but not of Group, WTS(2) = 0.00, *p* = 0.999. No significant Group × Time interaction was identified, WTS(2) = 0.27, *p* = 0.874. For Fitness, the analysis indicated a significant effect of Time, WTS(1) = 7.90, *p* = 0.005, but not of Group, WTS(2) = 2.54, *p* = 0.281. A significant Group × Time interaction was also identified, WTS(2) = 9.42, *p* = 0.009. The TGfU group showed a reduction in this dimension (*p <* 0.001, r = 0.808), whereas the Direct Instruction (*p* = 0.889, r = 0.030) and Comparison (*p* = 0.071, r = 0.394) groups did not differ significantly. For the Social dimension, the analysis indicated a significant effect of Time, WTS(1) = 48.60, *p <* 0.001, but no effect of Group, WTS(2) = 1.26, *p* = 0.533, nor a significant Group × Time interaction, WTS(2) = 2.72, *p* = 0.257.

**Table 1:** Results for motivation toward sport participation/physical education lessons.

MPAM-R Dimension	Intervention’s type					
	TGfU		Direct instruction		Comparison	
	Pre-test (M ± SD)	Post-test (M ± SD)	Pre-test (M ± SD)	Post-test (M ± SD)	Pre-test (M ± SD)	Post-test (M ± SD)
Enjoyment	**4.69 ± 0.81**	**6.42 ± 0.46** ^*^	6.06 ± 0.83	5.81 ± 0.80	5.94 ± 1.07	5.74 ± 1.32
Competence	**4.77 ± 1.06**	**5.99 ± 1.08** ^*^	5.72 ± 1.29	5.52 ± 1.43	5.24 ± 1.65	5.48 ± 1.64
Appearance	3.90 ± 1.62	3.46 ± 1.78	3.90 ± 1.44	3.46 ± 1.78	3.80 ± 1.69	3.56 ± 1.48
Fitness	**6.24 ± 1.21**	**5.55 ± 1.42** ^*^	5.63 ± 1.40	5.64 ± 1.45	5.82 ± 1.09	5.10 ± 1.62
Social	4.75 ± 0.80	4.12 ± 1.41	4.01 ± 1.47	4.02 ± 1.65	4.66 ± 1.64	4.45 ± 1.49

Bold values denote significant Group × Time interactions; ^*^Denotes large effect sizes.

### 3.2 Cognitive flexibility

Results for cognitive flexibility are presented in Table 2, based on performance on the Stroop Test. For the Word Reading variable, the analysis indicated a significant main effect of Time, WTS(1) = 7.96, *p* = 0.005. No main effect of Group was observed, WTS(2) = 1.96, p = 0.375, and no significant Group × Time interaction was identified, WTS(2) = 0.67, *p* = 0.714. For the Color Naming variable, the analysis indicated a significant main effect of Time, WTS(1) = 48.60, *p <* 0.001. No main effect of Group was observed, WTS(2) = 1.26, p = 0.533, and no significant Group × Time interaction was identified, WTS(2) = 5.10, *p* = 0.078.

**Table 2:** Stroop test results (cognitive flexibility).

Stroop test	Intervention’s type					
	TGfU		Direct instruction		Comparison	
	Pre-test (M ± SD)	Post-test (M ± SD)	Pre-test (M ± SD)	Post-test (M ± SD)	Pre-test (M ± SD)	Post-test (M ± SD)
Word reading	111.14 ± 1.90	111.90 ± 0.30	110.67 ± 2.06	111.05 ± 2.71	111.52 ± 0.81	111.76 ± 0.70
Color naming	70.43 ± 20.02	84.67 ± 16.35	68.48 ± 16.40	80.10 ± 16.55	77.14 ± 17.12	82.38 ± 19.83

### 3.3 Technical-tactical behavior

Technical-tactical indicators are presented in Table 3. For Accurate Passes, a significant main effect of Time was observed, WTS(1) = 5.74, *p* = 0.017, but no main effect of Group, WTS(2) = 1.35, *p* = 0.510. A significant Group × Time interaction was also identified, WTS(2) = 20.38, *p <* 0.001. *Post-hoc* analyses indicated a significant reduction from pre- to post-test in the Direct Instruction group (*p <* 0.001, r = 0.831), whereas no pre-post differences were observed in the TGfU (*p* = 0.585, r = 0.125) or Comparison groups (*p* = 1.000, r = 0.000). For Inaccurate Passes, a significant main effect of Time was found, WTS(1) = 10.76, *p* = 0.001, but not of Group, WTS(2) = 2.41, *p* = 0.299. In addition, the Group × Time interaction was significant, WTS(2) = 7.07, *p* = 0.029. The TGfU group showed a significant reduction in inaccurate passes (*p* = 0.002, r = 0.667), whereas the Direct Instruction (*p* = 0.659, r = 0.095) and Comparison (*p* = 0.082, r = 0.375) groups showed no change. Regarding Successful Receptions, no main effects of Time, WTS(1) = 2.34, *p* = 0.126, or Group, WTS(2) = 0.53, *p* = 0.766, were observed. However, a significant Group × Time interaction was identified, WTS(2) = 10.99, *p* = 0.004. Nevertheless, none of the *post-hoc* comparisons showed significant pre-post differences. For Unsuccessful Receptions, a significant main effect of Time was observed, WTS(1) = 7.49, *p* = 0.006, but not of Group, WTS(2) = 2.97, *p* = 0.226. No significant Group × Time interaction was found, WTS(2) = 4.02, *p* = 0.134.

**Table 3:** Technical analysis results.

Technical actions	Intervention’s type					
	TGfU		Direct instruction		Comparison	
	Pre-test	Post-test	Pre-test	Post-test	Pre-test	Post-test
	(M ± SD)	(M ± SD)	(M ± SD)	(M ± SD)	(M ± SD)	(M ± SD)
Accurate passes	15.10 ± 9.31	16.00 ± 6.33	**20.76 ± 8.90**	**10.14 ± 5.05** ^*^	13.81 ± 5.99	13.76 ± 7.76
Inaccurate passes	**1.71 ± 1.38**	**0.43 ± 0.75** ^*^	1.48 ± 2.11	1.05 ± 1.16	1.62 ± 1.32	0.95 ± 0.86
Successful receptions	15.76 ± 9.01	16.95 ± 5.95	20.57 ± 10.38	11.14 ± 6.51	15.14 ± 5.94	15.76 ± 8.45
Unsuccessful receptions	1.00 ± 0.84	0.57 ± 0.81	1.81 ± 1.83	0.76 ± 0.94	0.57 ± 0.68	0.62 ± 0.86
Goals scored	0.48 ± 0.68^#^	1.62 ± 1.32^#^	0.52 ± 0.87	0.57 ± 1.12	0.38 ± 0.59	0.90 ± 1.55
Shots off target	0.71 ± 1.38	0.62 ± 0.86	0.90 ± 1.37^#^	1.19 ± 1.03^#^	0.14 ± 0.36	0.71 ± 1.01

Bold values denote significant Group × Time interactions; Italic values denote significant main effects of Groups; ^#^denotes small effect sizes; ^*^denotes large effect sizes.

Analysis of Goals scored showed significant main effects of Time, WTS(1) = 4.14, *p* = 0.042, and Group, WTS(2) = 12.31, p = 0.002, indicating a higher frequency of shots on goal in the TGfU group compared with the Direct Instruction group (*p* = 0.022, r = 0.28) and the Comparison group (*p* = 0.036, r = 0.26). No significant Group × Time interaction was observed, WTS(2) = 5.71, *p* = 0.058. Finally, analysis of shots off target indicated significant main effects of Time, WTS(1) = 6.76, *p* = 0.009, and Group, WTS(2) = 6.15, *p* = 0.046, with a higher frequency of off-target shots in the Direct Instruction group compared with the Comparison group (*p* = 0.031, r = 0.26). No differences were observed between the TGfU group and the other groups (*p* > 0.05). No significant Group × Time interaction was found, WTS(2) = 1.71, *p* = 0.424.

#### 3.3.1 Offensive tactical actions

Analyses of offensive tactical actions (Table 4) are presented below based on the selected variables. For Off-ball movements to create passing options, significant main effects of Time were observed, WTS(1) = 12.61, *p <* 0.001, and Group, WTS(2) = 5.97, *p* = 0.050; however, *post-hoc* analyses did not identify statistically significant between-group differences (p > 0.05). A significant Group × Time interaction was also found, WTS(2) = 11.98, *p* = 0.003. Reductions were observed in the Direct Instruction (*p* = 0.012, r = 0.542) and Comparison (p = 0.006, r = 0.603) groups, but not in the TGfU group (*p* = 0.123, r = 0.334). For Movement after passing the ball, no main effects of Time, WTS(1) = 0.03, *p* = 0.859, or Group, WTS(2) = 3.38, *p* = 0.185, were observed. However, a significant Group × Time interaction was identified, WTS(2) = 6.33, *p* = 0.042. Only the TGfU group showed improvement (*p* = 0.039, r = 0.447), whereas the Direct Instruction (*p* = 0.137, r = 0.322) and Comparison (*p* = 0.474, r = 0.155) groups remained stable. For Occupying open spaces, a significant main effect of Time was observed, WTS(1) = 7.80, *p* = 0.005, but not of Group, WTS(2) = 5.32, *p* = 0.070. A significant Group × Time interaction was also found, WTS(2) = 6.66, *p* = 0.036. Only the TGfU group demonstrated a significant improvement (*p* = 0.0013, r = 0.698), whereas the Direct Instruction (*p* = 0.053, r = 0.421) and Comparison (*p* = 0.807, r = 0.053) groups showed no changes. For Shots on goal, no main effects of Time, WTS(1) = 2.77, *p* = 0.096, or Group, WTS(2) = 3.47, *p* = 0.176, were observed, and no significant Group × Time interaction was identified, WTS(2) = 0.53, *p* = 0.767.

**Table 4:** Offensive tactical actions results.

Tactical actions	Intervention’s type					
	TGfU		Direct instruction		Comparison	
	Pre-test (M ± SD)	Post-test (M ± SD)	Pre-test (M ± SD)	Post-test (M ± SD)	Pre-test (M ± SD)	Post-test (M ± SD)
Off-ball movements to create passing options	2.67 ± 2.59	2.90 ± 2.02	**2.86 ± 2.13**	**1.10 ± 1.58** ^*^	**3.10 ± 2.62**	**1.00 ± 1.70** ^*^
Movements after passing ball	**1.29 ± 1.62**	**2.14 ± 1.71** ^**+**^	1.48 ± 1.69	0.81 ± 1.12	2.00 ± 3.08	1.48 ± 2.60
Occupying open spaces	**1.19 ± 1.63**	**3.86 ± 3.65** ^*^	1.24 ± 1.48	3.10 ± 3.37	1.19 ± 1.44	1.33 ± 2.08
Shots on goal	2.38 ± 2.85	3.24 ± 2.61	3.29 ± 2.85	3.62 ± 3.01	1.81 ± 2.23	2.90 ± 2.91

Bold values denote significant Group × Time interactions; ^+^denotes medium effect sizes; ^*^denotes large effect sizes.

#### 3.3.2 Defensive tactical actions

Analyses of defensive tactical actions (Table 5) are presented below based on the selected variables. For Pressuring the ball carrier, no main effects of Time, WTS(1) = 0.27, *p* = 0.605, or Group, WTS(2) = 1.07, *p* = 0.586, were observed, and no significant Group × Time interaction was identified, WTS(2) = 1.83, *p* = 0.400. For Tracking an off-ball opponent, no main effects of Time, WTS(1) = 0.97, *p* = 0.326, or Group, WTS(2) = 2.89, *p* = 0.236, were observed. A significant Group × Time interaction was identified, WTS(2) = 6.83, *p* = 0.033; however, *post-hoc* analyses did not detect significant within-group pre-post differences: TGfU (*p* = 0.051, r = 0.425), Direct Instruction (*p* = 0.142, r = 0.319), and Comparison (*p* = 0.077, r = 0.379). For Interceptions, no main effects of Time, WTS(1) = 3.31, *p* = 0.069, or Group, WTS(2) = 0.16, *p* = 0.922, were observed. Nonetheless, a significant Group × Time interaction was found, WTS(2) = 9.11, *p* = 0.011. Only the Direct Instruction group showed improvement (p = 0.011, r = 0.554), whereas the TGfU (*p* = 0.712, r = 0.080) and Comparison (*p* = 0.575, r = 0.121) groups did not change. For Steals, no main effect of Time was observed, WTS(1) = 3.11, *p* = 0.078, and no significant Group × Time interaction was identified, WTS(2) = 1.99, *p* = 0.369. A significant main effect of Group was found, WTS(2) = 12.50, *p* = 0.002; however, *post-hoc* analyses did not identify statistically significant between-group differences (*p* > 0.081). For Shot blocks, no main effects of Time, WTS(1) = 0.47, *p* = 0.491, or Group, WTS(2) = 4.34, *p* = 0.114, were observed, and no significant Group × Time interaction was identified, WTS(2) = 1.03, *p* = 0.598.

**Table 5:** Defensive tactical actions results.

Defensive tactical actions	Intervention’s type					
	TGfU		Direct instruction		Comparison	
	Pre-test (M ± SD)	Post-test (M ± SD)	Pre-test (M ± SD)	Post-test (M ± SD)	Pre-test (M ± SD)	Post-test (M ± SD)
Pressuring the ball carrier	0.71 ± 0.85	1.24 ± 1.70	0.67 ± 0.86	1.14 ± 1.42	0.81 ± 0.93	0.62 ± 0.97
Tracking an off-ball opponent	0.71 ± 0.96	2.38 ± 3.04	0.71 ± 1.10	2.14 ± 3.32	1.33 ± 2.31	0.52 ± 1.21
Interceptions	1.05 ± 1.32	0.86 ± 1.01	**0.48 ± 1.08**	**1.62 ± 1.47** ^*^	1.29 ± 2.31	0.95 ± 1.40
Steals	0.24 ± 0.44	0.38 ± 0.67	0.20 ± 0.41	0.86 ± 1.46	0.05 ± 0.22	0.10 ± 0.30
Shot blocks	0.05 ± 0.22	0.14 ± 0.48	0.33 ± 0.73	0.24 ± 0.54	0.10 ± 0.44	0.19 ± 0.40

Bold values denote significant Group × Time interactions; ^*^ denotes large effect sizes.

## 4 Discussion

The present study examined whether participation in a TGfU-based handball unit was associated with changes in motivational, cognitive, technical, and tactical outcomes among middle-school students in a Brazilian public school. Overall, the findings partially supported the proposed hypotheses, showing that the TGfU group presented more favorable pre-post changes in selected motivational and tactical outcomes than the Direct Instruction and Comparison groups. However, given the cluster allocation with one class per condition and the absence of blinding, these findings should be interpreted as associations observed within this instructional context rather than as definitive evidence of the causal superiority of TGfU. The findings are consistent with previous investigations on the effects of TGfU in the context of school PE (Barba-Martín et al., 2020; Galeano-Rojas et al., 2023), as well as studies conducted specifically focused on handball teaching in Brazil, which have identified TGfU as a feasible and effective approach for promoting meaningful learning in school PE (Mazzardo et al., 2020; Mazzardo et al., 2022). Conversely, no significant between-group differences were observed for cognitive flexibility, a finding that extends the ongoing debate regarding the mechanisms underlying the pedagogical effects of TGfU, especially with respect to transfer to decontextualized cognitive measures.

From a motivational perspective, the hypothesis that the TGfU group would exhibit higher scores than the other groups was partially supported. Significant increases were observed only in the TGfU group for the Enjoyment and Competence dimensions, both of which are linked to intrinsic motivation within Self-Determination Theory (Deci and Ryan, 2000). These results suggest that the centrality of gameplay, a defining feature of TGfU, promotes more enjoyable experiences and higher perceived competence among students. This pattern aligns with previous evidence indicating that TGfU can foster greater engagement, autonomy, and satisfaction of basic psychological needs in school PE (Barba-Martín et al., 2020; Gil-Arias et al., 2017; Ortiz et al., 2023). The use of modified and small-sided games, coupled with continuous tactical problem solving mediated through pedagogical questioning, likely increases students’ active involvement and perceived success, which are key drivers of intrinsic motivation. It is noteworthy that the MPAM-R, as an instrument assessing reasons (motives) for physical activity participation, is not a direct measure of the psychological need satisfaction constructs central to the Self-Determination Theory (i.e., autonomy, competence, and relatedness as needs rather than as motivational outcomes). The MPAM-R, by contrast, captures the downstream motivational consequences of need satisfaction, particularly enjoyment and perceived competence, rather than the need satisfaction process itself (Ryan et al., 1997; Albuquerque et al., 2017). In the present study, Self-Determination Theory serves as a broad interpretive frame: the improvements in Enjoyment and Competence in the TGfU group are consistent with the prediction that autonomy-supportive, game-centered learning environments satisfy the needs for autonomy and competence (and potentially relatedness), thereby fostering more intrinsically oriented motivational states. However, because the instrument does not directly measure need satisfaction, this interpretation remains inferential.

In contrast, no significant changes were observed in the Appearance and Social dimensions, and a reduction was detected in the Fitness dimension in the TGfU group. This pattern may reflect a shift in students’ motivational focus, with greater value being placed on enjoyment and game learning rather than instrumental goals related to physical fitness. Moreover, the general increase in motivational scores over time across all groups suggests that, although the instructional model is relevant, it does not operate in isolation in shaping the motivational climate of lessons. Evidence indicates that contextual factors, such as the teacher’s climate, characterized by relatedness support, emphasis on effort, and acceptance of errors as part of the learning process, exert a meaningful influence on students’ motivation (Chen et al., 2020). In addition, participants’ lack of prior handball experience may have amplified perceptions of progress and competence throughout the intervention, regardless of the adopted model (Carcamo-Oyarzun et al., 2025).

Because cognitive flexibility was assessed exploratorily using a decontextualized proxy (Stroop test), the absence of group differences should not be interpreted as evidence that TGfU lacks cognitive demands. Rather, the findings indicate that the observed time-related improvements in Stroop performance were not specific to the TGfU condition. These results indicate that, despite TGfU involving decision-making demands in dynamic and unpredictable environments (Robles et al., 2020; González-Valero et al., 2024), such experiences may not be sufficient to elicit measurable transfer to decontextualized cognitive tests. The literature suggests that consistent changes in executive functions, such as cognitive flexibility, may require longer interventions, with more systematic manipulation of task complexity and cognitive load (Lee et al., 2024). Thus, the cognitive gains observed for all the groups across time may be more strongly related to sustained participation in team sports than to the specific pedagogical model, as indicated by prior work (Kolovelonis et al., 2022; Kolovelonis and Goudas, 2023).

Regarding technical outcomes, the hypothesis that the handball groups would show more favorable changes than the Comparison group was only partially supported. The TGfU group showed a reduction in inaccurate passes, suggesting greater functional efficiency in maintaining possession during gameplay. In contrast, the Direct Instruction group showed a reduction in accurate passes, suggesting difficulty in transferring technique-focused practice to dynamic game situations. These findings should be interpreted cautiously, but they are consistent with the view that early learning in invasion games depends not only on isolated execution but also on the contextual use of technical actions under tactical constraints. This pattern is consistent with contemporary motor and cognitive learning accounts, which describe behavioral variability as a typical feature of early learning phases, preceding the stabilization of technical efficiency (Hossner and Zahno, 2022; López-Fernández et al., 2025).

The most robust effects of the study were observed in tactical behavior, particularly in offensive actions performed without ball possession. The TGfU group demonstrated consistent improvements in behaviors such as movement after passing, occupying open spaces, getting free from defenders, and penetrating toward the goal. In contrast, the Direct Instruction and Comparison groups remained stable or showed reductions. These findings suggest that TGfU appears to facilitate the operational principles of invasion games, such as maintaining possession, advancing toward the target, and achieving offensive superiority, through representative learning tasks and collective tactical problem-solving (Bayer, 1994). The number of significant improvements observed in these measures from pre to post-test could also be explained by the fact that these actions were performed without the ball, reducing the motor demands associated with ball handling and allowing participants to focus more on tactical movement and positioning. Recent evidence further indicates that game-based approaches promote greater cognitive engagement and decision-making in off-ball offensive actions, which are fundamental to functional game performance in school contexts (Sierra-Ríos et al., 2020; González-Valero et al., 2024).

Although the findings presented earlier generally corroborate the results of recent literature reviews demonstrating positive effects of TGfU on decision-making and skill execution (Barba-Martín et al., 2020; Galeano-Rojas et al., 2023; Ortiz et al., 2023), it is important to approach these findings with a critical perspective, as the effectiveness of TGfU is influenced by a variety of implementation, learner, and contextual factors (Breed et al., 2025). While TGfU led to improvements in tactical behaviors, such as off-ball movement and positioning, it is important to note that these gains may come at the expense of technical precision, particularly in the short term. The lack of effects on technical variables like accurate passes, which actually declined in the Direct Instruction group, could be partly explained by the floor effects resulting from students’ lack of prior experience with handball. In the early stages of skill acquisition, both TGfU and DI may face limitations: DI may yield higher performance in controlled, technical drills, but its impact on game-like situations is more limited (Stolz and Pill, 2014). In contrast, TGfU, while promoting tactical development, may not lead to immediate technical improvements, but it fosters a more comprehensive understanding of game dynamics. These considerations reinforce the need for longitudinal studies that track both technical and tactical development over time, offering a more nuanced view of the long-term impact of each model.

For defensive actions, results were more heterogeneous. The improvement observed in the TGfU group in defensive coverage suggests greater development of collective defensive behaviors that depend on game reading and coordinated teammate action. Conversely, the increase in interceptions in the Direct Instruction group indicates that more analytical approaches may foster specific reactive defensive actions. The high-repetition, low-variability drills focused on predictable pass-and-receive sequences likely helped students develop pattern recognition for pass trajectories, enhancing their ability to anticipate passes in controlled settings, but limiting passing ability transfer to more dynamic, game-like contexts. These patterns reflect the well-documented skill specificity of blocked practice and the limited transfer of drill-based learning to representative game situations (Robles et al., 2020; Stolz and Pill, 2014). Overall, these findings support the notion that different pedagogical models may preferentially develop distinct components of defensive behavior, with collective actions being more sensitive to approaches centered on tactical understanding and individual actions being more closely associated with structured, teacher-directed practice (Sierra-Ríos et al., 2020; González-Espinosa et al., 2021; Ortiz et al., 2023).

The present findings carry several concrete implications for PE teachers and curriculum designers, particularly for teaching handball in the Brazilian public school context. First, the results suggest that a 20-lesson TGfU-based handball unit is feasible in an authentic Brazilian public school setting and produces meaningful gains in intrinsic motivation and tactical game behavior. Additionally, the significant decline in accurate passes in the Direct Instruction group is another practically important finding for teachers and program coordinators. It suggests that a high proportion of lesson time spent on technique-isolated practice does not guarantee transfer to authentic game performance, and may even disrupt the game-action fluency that novice learners develop when allowed to practice within representative game contexts (Robles et al., 2020). These findings are relevant for teachers who may hesitate to adopt game-based models with beginners, fearing insufficient structure or slow technical progress: the present data indicate that TGfU can support motivational engagement and tactical development in handball even with students who have no prior experience in the sport, provided that lessons are organized around appropriately modified small-sided games and systematic tactical questioning.

The results also reinforce the importance of teacher training in game-based models. The successful implementation of TGfU in the present study depended on the teacher’s capacity to design modified game formats, formulate tactical questions, and manage the cognitive complexity of the learning environment. In most Brazilian public schools, these competencies are not systematically developed in pre-service or in-service teacher education (Mazzardo et al., 2022; Arantes et al., 2025). Investment in professional development programs that train PE teachers in game-based pedagogy including practice with modified game design and questioning techniques is therefore a critical complementary action if TGfU’s motivational and tactical benefits are to be replicated at scale in the Brazilian context.

Finally, the students’ lack of prior experience likely contributed to the observed improvements in motivational and tactical behaviors, making these findings particularly relevant for beginner or novice learners. However, these results should not be directly generalized to more experienced students, as the impact of TGfU may differ depending on the level of prior knowledge and skill in the sport. Therefore, teachers should approach model selection with a clear understanding of the specific learning objectives of each unit, the characteristics of the student population, and the pedagogical conditions available in their institutional context.

### 4.1 Limitations

This study has limitations that should be considered when interpreting the findings. First, allocation occurred at the class level, with one class per arm; therefore, class-level influences, including classroom dynamics, cannot be fully disentangled from the instructional condition. Second, although the same teacher delivered all three units, thereby reducing between-teacher variability, the teacher was not blinded to group allocation, and personal familiarity with one model may have influenced implementation. Third, outcome assessment was not blinded, which may have introduced assessment bias. Fourth, although students reported no structured experience in handball or other collective sports, informal exposure to other invasion games could not be fully controlled and may have influenced tactical indicators, particularly off-ball movement, occupation of open spaces, and defensive positioning. Fifth, statistical power was moderate for some tactical outcomes, likely because tactical behavior during small-sided games is inherently variable. Finally, the absence of spatial-tracking and physical-load measures limited deeper analysis of how students occupied, created, and defended space during gameplay. Future studies should include multiple classes per arm, blinded performance coding, and multimodal assessment to strengthen causal inference and clarify the pedagogical mechanisms underlying students’ motivational and game-performance responses.

## 5 Conclusion

In summary, implementing a TGfU-based handball unit in a Brazilian public-school context was associated with meaningful improvements in motivational outcomes (particularly enjoyment and perceived competence) and in several technical-tactical behaviors during small-sided games. Pedagogically, these findings reinforce the value of game-centered models for invasion sports because they align learning tasks with the logic of the game: modified/representative games, manipulation of constraints (space, rules, player numbers), and structured questioning appear to promote students’ tactical problem solving and off-ball involvement, dimensions that are difficult to cultivate through technique-first instruction alone.

From a methodological perspective in sport pedagogy, the pattern of results supports the importance of ecologically valid assessment. When learning is evaluated through game-based performance indicators, the TGfU condition was associated with functional changes consistent with the demands of real play. At the same time, the absence of differential effects on cognitive flexibility (Stroop) indicates that cognitively rich gameplay does not necessarily translate to improvements on decontextualized executive-function tests within this dosage and setting, underscoring the need to consider the specificity of transfer and measurement sensitivity. Given the students’ lack of prior experience with handball, the cluster allocation with one class per condition and the lack of blinded assessment, conclusions should be interpreted cautiously. Future studies should include multiple classes per arm, blinded performance coding, and multimodal outcomes (psychological need satisfaction, teacher interpersonal style, and game-performance micro-indicators) to better isolate pedagogical mechanisms and clarify how motivational climates and task constraints jointly shape learning in school sport.

## Data Availability

The raw data supporting the conclusions of this article will be made available by the authors, without undue reservation.
