# Supplementary material for: Teaching games for understanding in school handball: a controlled pre-post study with intact Brazilian physical education classes
Source: Front Psychol. 2026 May 19;17:1817709. doi: 10.3389/fpsyg.2026.1817709 (PMC13226001; doi:10.3389/fpsyg.2026.1817709)
Supplement: Supplementary file 1 [file Table_1.DOCX]

# Supplementary Material 1. Didactic Unit - 20 Lessons

# Game formats progress in complexity across the unit for all groups. Broad lesson objectives served as pedagogical organizers throughout the unit, whereas specific content and learning tasks varied according to the instructional model and sport modality.

| **Lesson** | **Broad Lesson Objectives (pedagogical organizers)** | **TGfU Group - Handball** | **Direct Instruction - Handball** | **Direct Instruction - Volleyball** |
| --- | --- | --- | --- | --- |
|  | **ATK / DEF** | **Content + Tactical Principles + Questions + Game Format** | **Content + Game Format** | **Content + Game Format** |
| **1** | **ATK:** Ball possession maintenance;  **DEF:** Close passing lines; individual marking | **ATK:** Create passing lines; find open spaces to receive  **DEF:** Close passing lines; stay between your player and the ball  **Principle:** Representation; Exaggeration (3v1 overload - high success rate for beginners)  **Tactical Questions:**  • Where do you need to stand so your teammate can pass to you easily?  • What happens when you stay too close to the ball carrier?  • If you were the defender, where would you stand to make the pass harder?  **Game format:** 2v2 (no goalkeeper) - cone goals 2m wide | **ATK:** Basic overarm throw technique: grip, stance, step-and-throw, wrist snap  **DEF:** Defensive ready stance: feet shoulder-width, knees bent, weight forward, one arm raised  **Game format:** 4v4 + goalkeeper - cone goals | **ATK:** Overhead set: hand triangle, contact point on finger pads, upward push motion.  **DEF:** Defensive ready position: bent knees, feet shoulder-width, weight on balls of feet; side-step movement  **Game format:** 4v4 across low net (1.8m) |
| **2** | **ATK:** Create space to receive the ball; ball possession maintenance  **DEF:** Close passing lines; individual marking; begin to understand goal defence | **ATK:** Move before receiving; create passing lines through off-ball movement  **DEF:** Close passing lines; individual marking responsibility  **Principle:** Exaggeration (no movement carrying the ball rule forces off-ball movement); Representation  **Tactical Questions:**  • Why is it better to move before your teammate passes to you?  • Where did you move to make it easier to receive the ball?  • As a defender, what did you do when the attacker moved away from you?  **Game format:** 2v2 (no goalkeeper) - cone goals 2m wide | **ATK:** Passing to a moving target; timing the pass; pass-and-move pattern  **DEF:** Closing passing lines: positioning between two attackers to intercept  **Game format:** 4v4 + goalkeeper | **ATK:** Overhead set: hand triangle, contact point on finger pads, upward push motion.  **DEF:** Defensive ready position: bent knees, feet shoulder-width, weight on balls of feet; side-step movement  **Game format:** 4v4 across low net |
| **3** | **ATK:** Choose the safest pass; ball possession maintenance; first shots at goal  **DEF:** Close passing lines; individual marking; begin to understand defensive protection of the scoring area | **ATK:** Decide between passing and shooting based on defensive positioning; choose the safe option  **DEF:** Close passing lines; block the shooting lane  **Principle:** Representation (goal introduced in safe overload context); Exaggeration (3v1 maintains high success)  **Tactical Questions:**  • When is it safer to pass - and when is it a good moment to shoot?  • What did you look at to decide whether to pass or shoot?  • If you were the defender, how did you try to stop the shot?  **Game format:** 2v2 + goalkeeper - first contact with goalkeeper role | **ATK:** Shooting technique: overarm throw at goal; step-and-throw footwork pattern; contact point  **DEF:** Individual 1v1 marking footwork: mirror movement, staying between attacker and goal  **Game format:** 4v4 + goalkeeper | **ATK:** Overhead set: hand triangle, contact point on finger pads, upward push motion.  **DEF:** Defensive ready position: bent knees, feet shoulder-width, weight on balls of feet; side-step movement  **Game format:** 4v4 across low net |
| **4** | **ATK:** Create passing lines; occupy different spaces in attack  **DEF:** Close passing lines; individual marking responsibility; protect the scoring area | **ATK:** Spread out across the court; create passing lines by occupying different spaces  **DEF:** Close passing lines; individual marking - each defender responsible for one attacker  **Principle:** Exaggeration (4v2 overload + corner-cone rule makes spreading out visible and achievable); Representation  **Tactical Questions:**  • Why did the teacher ask you to start near different corners?  • What happened when two teammates stood in the same area?  • As a defender, which attacker were you responsible for - and how did you decide?  **Game format:** 2v2 + goalkeeper | **ATK:** Pass-and-move: passing to a moving target; immediate movement after passing  **DEF:** Defensive sliding: two defenders moving together to block passing lanes without crossing feet  **Game format:** 4v4 + goalkeeper | **ATK:** Overhead set: hand triangle, contact point on finger pads, upward push motion.  **DEF:** Defensive ready position: bent knees, feet shoulder-width, weight on balls of feet; side-step movement  **Game format:** 4v4 across low net |
| **5** | **MOTOR SKILL SESSION**  Skill refinement | **ATK:** Ball handling; grip and release mechanics; receiving while moving  **DEF:** Defensive footwork; ready position; hand-eye coordination  **Principle:** Representation (all skills practiced within game - not in isolation)  **Tactical Questions:**  • How do you hold the ball before you throw it - and why does it matter?  • What happens to the pass when your wrist snaps at the end?  **Game format:** 3v3 (no goalkeeper) - conditioned game; skills practiced in game context | **ATK:** Overarm throw; catch-and-pivot; dribbling in a straight line; receiving a rolling ball  **DEF:** Defensive reaction; lateral shuffle; jump reach (blocking simulation)  **Game format:** 4v4 + goalkeeper | **ATK:** Forearm pass technique: platform position, angle of contact, ball direction control  **DEF:** Defensive ready position: bent knees, feet shoulder-width, weight on balls of feet; side-step movement  **Game format:** 4v4 across low net |
| **6** | **GAME EVALUATION**  Performance Assessment | **ATK:** Application of ball possession and first finishing concepts in game context  **DEF:** Individual marking; closing passing lines; basic goal protection  **Principle:** Assessment: focus on observing support movement (ATK) and marking position (DEF)  **Tactical Questions:**  • Who found the most open spot today - and how did they do it?  • When your team lost the ball, what did the nearest player do first?  **Game format:** 3v3 + goalkeeper | **ATK:** Application of passing technique and pass-and-move in game; shooting from reasonable distance  **DEF:** Defensive stance and positioning; staying between opponent and goal  **Game format:** 4v4 + goalkeeper - evaluation | **ATK:** Forearm pass technique: platform position, angle of contact, ball direction control  **DEF:** Defensive ready position: bent knees, feet shoulder-width, weight on balls of feet; side-step movement  **Game format:** 4v4 across low net |
| **7** | **ATK:** Offensive progression and space use  **DEF:** Prevent or delay opponent's progression; understand collective defensive shape | **ATK:** Move forward with the ball; exploit numerical advantage (3v2); support the ball carrier in attack  **DEF:** Delay the attack; do not both defenders commit to the ball simultaneously  **Principle:** Exaggeration (3v2 overload makes forward progression achievable for beginners); Representation  **Tactical Questions:**  • When you had 3 players vs. 2 defenders, who ran toward the goal and who stayed wide?  • Defenders: what happened when both of you went to the same attacker?  • If you are the last defender, do you go for the ball or slow the attacker down - and why?  **Game format:** 3v3 + goalkeeper | **ATK:** Shooting on the move: 1–2 steps after reception; legal footwork pattern before throwing  **DEF:** Defensive retreat: sprint to defensive position before attacker reaches the goal zone  **Game format:** 4v4 + goalkeeper | **ATK:** Forearm pass technique: platform position, angle of contact, ball direction control  **DEF:** Defensive ready position: bent knees, feet shoulder-width, weight on balls of feet; side-step movement  **Game format:** 4v4 across low net |
| **8** | **ATK:** Offensive progression in depth  **DEF:** Prevent and/or delay opponent's progression; begin to protect the scoring area collectively | **ATK:** Move the ball forward through zones; support runs in depth; follow the ball into attack  **DEF:** Prevent progression into the next zone; each defender covers their zone  **Principle:** Exaggeration (zone-based scoring makes forward progression the visible objective); Representation  **Tactical Questions:**  • Why is it important to move forward when your team has the ball?  • When you passed the ball forward, what did you do next - stand still or follow the ball?  • Defenders: which zone was hardest to protect and why?  **Game format:** 3v3 + goalkeeper | **ATK:** Passing in depth: timing the forward pass; support runs alongside the ball carrier  **DEF:** Defensive shape and retreat: 2-player defensive line; delay tactics without committing  **Game format:** 5v5 + goalkeeper | **ATK:** Forearm pass technique: platform position, angle of contact, ball direction control  **DEF:** Defensive ready position: bent knees, feet shoulder-width, weight on balls of feet; side-step movement  **Game format:** 5v5 across low net |
| **9** | **ATK:** Offensive progression using depth and width  **DEF:** Prevent and/or delay opponent's progression; protect the scoring area | **ATK:** Exploit width and depth simultaneously; pass to wide spaces before shooting  **DEF:** Prevent entry into wide corridors AND prevent shots at goal; defend as a group  **Principle:** Exaggeration (bonus for using wide corridor rewards use of width - counteracts natural central crowding); Representation  **Tactical Questions:**  • Why did the teacher give a bonus point for passing from the sides?  • When does using the wide corridor make the shot on goal easier?  • Defenders: where did you have to move when an attacker went wide?  **Game format:** 3v3 + goalkeeper | **ATK:** Width-and-depth passing: switching the point of attack; passing wide before advancing  **DEF:** Collective defensive structure: 3-player defensive line; lateral movement as a unit  **Game format:** 5v5 + goalkeeper | **ATK:** Alternating forearm pass and overhead set: controlling the ball between two contact types  **DEF:** Block placement: hand position above net; stance before jumping; no spike yet  **Game format:** 5v5 across low net |
| **10** | **ATK:** Maintain offensive continuity, progress, and finish  **DEF:** Recover possession; prevent progression; protect the scoring area | **ATK:** Transition to attack after winning the ball; reorganize possession before advancing forward  **DEF:** Recover possession; gentle pressure on ball carrier  **Principle:** Representation (transition mimics real game recovery); Exaggeration (mandatory backward pass gives time to reorganize)  **Tactical Questions:**  • Why did the rule ask you to pass backward first after winning the ball?  • After your team won the ball, who was the first person to call for it?  • As a defender, how close do you stand to the ball carrier when trying to win the ball back?  **Game format:** 3v3 + goalkeeper | **ATK:** Ball recovery and transition to attack: recognizing the moment to go forward  **DEF:** Pressing: hands-up stance and footwork pressure; no grabbing  **Game format:** 5v5 + goalkeeper | **ATK:** Alternating forearm pass and overhead set: controlling the ball between two contact types  **DEF:** Block placement: hand position above net; stance before jumping; no spike yet  **Game format:** 5v5 across low net |
| **11** | **MOTOR SKILL SESSION**  Skill refinement | **ATK:** Passing; receiving while moving; dribbling; shooting at goal  **DEF:** Defensive footwork; lateral movement; marking stance  **Principle:** Representation (all skills embedded in game context)  **Tactical Questions:**  • When did you choose to dribble instead of pass - what made you decide?  • What is harder: dribbling while standing still or dribbling while moving forward?  **Game format:** 3v3 + goalkeeper - conditioned (team must involve 2 players before scoring) | **ATK:** Dribbling in a straight line; bounce pass; overarm throw at target; catching while moving sideways  **DEF:** Mirror drill; lateral shuffle; turn-and-sprint reaction  **Game format:** 5v5 + goalkeeper | **ATK:** 3-touch sequence introduction: forearm pass → overhead set → send over; sequence before result  **DEF:** Blocker-and-cover partnership: positioning, communication, and responsibility areas at the net  **Game format:** 5v5 across low net |
| **12** | **GAME EVALUATION**  Performance Assessment | **ATK:** Decisions in possession and progression; support movement off the ball  **DEF:** Individual marking; marking position relative to ball and goal  **Principle:** Game simplified observation: support movement (ATK) and marking position (DEF)  **Tactical Questions:**  • Was there a moment when you had a clear shot - what did you decide to do?  • Did you always know which player you were marking? How did you keep track?  **Game format:** 4v4 + goalkeeper - evaluation game | **ATK:** Overarm throw technique in game; movement after passing; shooting from ≤ 8m  **DEF:** Defensive stance; positioning relative to ball and goal  **Game format:** 5v5 + goalkeeper - evaluation | **ATK:** 3-touch sequence introduction: forearm pass → overhead set → send over; sequence before result  **DEF:** Blocker-and-cover partnership: positioning, communication, and responsibility areas at the net  **Game format:** 5v5 across low net - evaluation |
| **13** | **ATK:** Integration of key offensive principles  **DEF:** Recover possession; prevent progression; protect the scoring area (first full defensive integration) | **ATK:** Combine all three offensive principles in sequence: possession → midline crossing → finish  **DEF:** Recover possession; prevent midline crossing; prevent shots  **Principle:** Exaggeration (double-point incentive for complete attack chain makes the full sequence visible); Representation  **Tactical Questions:**  • What did your team do first after winning the ball - and was that the best choice?  • Which part of the attack sequence was easiest: keeping the ball, moving forward, or shooting?  • Defenders: when did you all need to work together instead of just individually?  **Game format:** 4v4 + goalkeeper | **ATK:** Full attack combination: ball retention (2 passes) → dribble progression → support run → shot  **DEF:** Two-defender combination: one on ball, one covering second attacker  **Game format:** 5v5 + goalkeeper | **ATK:** Attack approach mechanics: 3-step approach, jump for height; no ball contact yet  **DEF:** Block footwork: side-step to position at net; read ball direction before jumping  **Game format:** 5v5 across low net |
| **14** | **ATK:** Decision-making in attack according to sport-specific game situations  **DEF:** Recover possession; prevent progression; protect the scoring area; individual marking responsibility | **ATK:** Make explicit decisions: pass, dribble, or shoot; read the defender to choose the correct option  **DEF:** Individual marking; recover possession through footwork only  **Principle:** Representation; Exaggeration (freeze moments make decision-making explicit and discussable for beginners)  **Tactical Questions:**  • When the game was frozen - you had the ball and a defender was close - what were your options?  • Which clue tells you it is a good moment to shoot rather than pass?  • After winning the ball, what is the first thing you should look for?  **Game format:** 4v4 + goalkeeper | **ATK:** Attacking against different stance defenders (passive blocker in 6m, active marker in 9m, etc.)  **DEF:** Individual active marking: hands-up stance; slide to stay between attacker and goal  **Game format:** 5v5 + goalkeeper | **ATK:** Attack approach mechanics: 3-step approach, jump for height; ball contact  **DEF:** Block-and-cover combination: positioning and communication between blocker and cover player  **Game format:** 5v5 across low net |
| **15** | **ATK:** Progression and finishing; decision-making; support runs (off-ball movement to help a teammate)  **DEF:** Recover possession; prevent progression; protect the scoring area | **ATK:** Support runs: move to a new space before asking for the ball; create 2v1 situations through movement  **DEF:** Recover possession; prevent shots; individual marking  **Principle:** Exaggeration (designated runner role makes support runs concrete and observable for beginners); Representation  **Tactical Questions:**  • When your teammate had the ball, where did the runner go - toward the ball or away from it?  • Why is it helpful when a teammate runs to a new spot before asking for the ball?  • Defenders: what did you do when you saw the runner making their move?  **Game format:** 4v4 + goalkeeper | **ATK:** Give-and-go (simplified wall pass): pass, then immediately relocate to a new space for the return  **DEF:** Two-defender coverage: one presses ball carrier; other covers second attacker 2m behind  **Game format:** 5v5 + goalkeeper | **ATK:** Attack approach mechanics: 3-step approach, jump for height; ball contact  **DEF:** Block-and-cover combination: positioning and communication between blocker and cover player  **Game format:** 5v5 across low net |
| **16** | **ATK:** Integration of key offensive principles throughout play  **DEF:** Individual marking; recover possession; prevent progression; protect the scoring area | **ATK:** Full integration: maintain possession → advance midline → support run touch → finish at goal  **DEF:** Individual marking; group defensive shape; protect goal  **Principle:** Exaggeration (sequential 3-point scoring makes each principle visible simultaneously); Representation (closest to real game yet)  **Tactical Questions:**  • Which part of the 3-point sequence was hardest for your team to complete - and why?  • How did your team's communication change when trying for 3 points vs. just 1?  • Defenders: which moment in the opponent's attack was easiest to interrupt - and how?  **Game format:** 4v4 + goalkeeper | **ATK:** Full attack sequence: 2 passes in back zone → 1 advancing dribble → shoot from inside 9m; rotating finishing role  **DEF:** Full defensive shape: individual assignment + collective retreat + marking position  **Game format:** 6v6 + goalkeeper | **ATK:** Serve-receive-to-attack chain: 3-touch sequence from reception to attack finish  **DEF:** Dig recovery: return to ready position immediately after defensive contact  **Game format:** 6v6 across low net |
| **17** | **MOTOR SKILL SESSION**  Skill refinement | **ATK:** Passing; reception on the move; shooting at goal  **DEF:** Defensive footwork; marking awareness  **Principle:** Representation (skills refined only within game - no isolated drilling)  **Tactical Questions:**  • How do you know your teammate is ready to receive your pass?  • What changed in your throwing compared to Lesson 1 - what is better now?  **Game format:** 4v4 + goalkeeper - conditioned (goal counts only if preceded by a moving-player assist) | **ATK:** Overarm throw accuracy; bounce pass relay; dribble + 2-step shoot; pass-and-sprint receive  **DEF:** Mirror drill; defensive stance hold; sprint-and-recover between cones  **Game format:** 6v6 + goalkeeper | **ATK:** Serve-receive-to-attack chain: 3-touch sequence from reception to attack finish  **DEF:** Dig recovery: return to ready position immediately after defensive contact  angle correction; reaction-and-dig on teacher's call  **Game format:** 6v6 across low net |
| **18** | **GAME EVALUATION**  Performance Assessment | **ATK:** Decision-making quality (pass/shoot choice); support movement (off-ball positioning)  **DEF:** Individual marking; collective goal protection  **Principle:** Formative game evaluation; game-based observation  **Tactical Questions:**  • Was your team better at keeping the ball, moving forward, or shooting - what was hardest?  • Which defensive moment stopped an attack effectively - what happened?  **Game format:** 4v4 + goalkeeper - evaluation game | **ATK:** Technique quality in passing, dribbling, and shooting; movement after passing  **DEF:** Marking position and defensive transitions  **Game format:** 6v6 + goalkeeper - evaluation | **ATK:** Serve-receive-to-attack chain: 3-touch sequence from reception to attack finish  **DEF:** Dig recovery: return to ready position immediately after defensive contact  **Game format:** 6v6 across regular net |
| **19** | **ATK:** Progression + finishing; support runs - consolidation  **DEF:** Recover possession; prevent progression; protect the scoring area-consolidation | **ATK:** Consolidate the support-run principle: assist from a running player earns a bonus; cooperative attack  **DEF:** Individual marking; recover possession; protect goal  **Principle:** Exaggeration (assist bonus reinforces cooperative attack principle); Representation (full game)  **Tactical Questions:**  • How many of your goals today were 2-pointers (assist from a running player)?  • Compared to the initial lessons, does your team move more now - what changed?  • Which defensive move stopped an attack in the last 5 minutes?  **Game format:** 4v4 + goalkeeper | **ATK:** Offensive transition passing and dribbling in real-speed overload situations  **DEF:** 2-defender combination: pressure on ball + cover of second attacker  **Game format:** 6v6 + goalkeeper | **ATK:** Full 3-touch chain at controlled pace: serve-receive → set → attack  **DEF:** Block-and-defense: blocker at net + two defenders players reading the attack  **Game format:** 6v6 across regular net |
| **20** | **FINAL GAME EVALUATION**  Performance Assessment | **ATK:** Application of key offensive principles; decision-making across all game situations  **DEF:** Full defensive integration: individual marking + collective shape + defensive protection of the scoring area  **Principle:** Game evaluation; post-unit individual feedback and group reflection  **Tactical Questions:**  • Looking back at 20 lessons - what is the biggest change you notice in how you play?  • Which principle - keeping the ball, moving forward, or finishing - feels most natural now?  **Game format:** 4v4 + goalkeeper – evaluation game | **ATK:** Full technical and tactical performance: passing quality, movement, shooting, decision-making  **DEF:** Defensive stance, marking position, and recovery behaviour  **Game format:** 6v6 + goalkeeper | **ATK:** Full 3-touch chain at controlled pace: serve-receive → set → attack  **DEF:** Block-and-defense: blocker at net + two defenders players reading the attack  **Game format:** 6v6 across regular net |

ATK = Offensive content; DEF = Defensive content; GK = Goalkeeper; TGfU game format progresses from 2v2+GK (Lessons 1–4) → 3v3+GK (Lessons 5–12) → 4v4+GK (Lessons 13–20). DI-Handball progresses from 4v4+GK (Lessons 1–7) → 5v5+GK (Lessons 8–15) → 6v6+GK (Lessons 16–20). DI-Volleyball progresses from 4v4 (Lessons 1–7) → 5v5 (Lessons 8–15) → 6v6 (Lessons 16–20). Specific learning tasks were designed according to the instructional model and sport modality.
