# Supplementary material for: Teaching games for understanding in school handball: a controlled pre-post study with intact Brazilian physical education classes
Source: Front Psychol. 2026 May 19;17:1817709. doi: 10.3389/fpsyg.2026.1817709 (PMC13226001; doi:10.3389/fpsyg.2026.1817709)
Supplement: Supplementary file 2 [file Table_2.DOCX]

Supplementary Material 2. Lesson plan examples (Lesson 4)

**Shared lesson objectives (all groups).**

**Lesson 4 - TGfU Group (Handball)**

**Operational Principles (Bayer, 1994)**

• ATK - Ball possession maintenance: create passing lines by occupying different spaces

• DEF - Ball recovery: close passing lines to prevent clean reception

• DEF - Goal protection: individual marking responsibility (one defender per attacker)

**Lesson Objectives**

• ATK: Spread out across the court; occupy different spaces; create passing lines through off-ball movement

• DEF: Close passing lines; each defender is individually responsible for one attacker

| **Phase** | **Time** | **Description** |
| --- | --- | --- |
| **Warm-Up** | 8 min | **3 vs. 1 Keep-Away (Review from Lesson 3)**  • Organization: groups of 4; one ball per group; square ~7m × 7m marked with cones.  • Task: 3 attackers keep the ball away from 1 defender; the defender who intercepts or forces an error swaps with the attacker who made the poorest pass. Passing above the defender is not allowed.  • Transition cue: "Now we are going to make it harder - 2 defenders." |
| **Main Activity** | 28 min | **4 vs. 2 Keep-Away with Corner-Cone Rule**  Organization:  • Court: ~9m × 9m square; 1 cone at each corner.  • Teams: 4 attackers vs. 2 defenders; rotating defenders every 2 minutes (or immediately after interception/forced error).  • Starting rule: each attacker must begin near a different corner cone.  • Scoring: attackers earn 1 point for every 5 consecutive successful passes; defenders earn 1 point per interception. Passing above the defenders is not allowed.  Stop-and-freeze moments (teacher pauses game 2–3 times):  • Situation 1 - two attackers crowd the same zone:  – "Look at where you and your teammate are standing - are you helping each other or getting in each other's way?"  – Expected insight: clustering reduces passing options; spreading forces defenders to cover more ground.  • Situation 2 - attacker stands still after receiving:  – "You just received the ball. What could you have done before it arrived to make things easier for yourself?"  – Expected insight: moving before the pass is what creates the passing line.  • Situation 3 - ask a defender:  – “Which attacker were you responsible for - and how did you position yourself to mark them?”  – Expected insight: individual marking means one defender, one attacker; trying to keep close distance to the attacker at all times. |
| **Collective Game** | 9 min | **2 vs. 2 + Goalkeeper - Cone Goals (2m wide)**  • Organization: court ~15m × 8m; cone goals at each end; student goalkeeper rotates every 3 minutes.  • Rules: overarm pass only; ≤ 3 seconds in possession; no physical contact.  • No rule modifications to the game - authentic small-sided experience with lesson principles in a free context. |
| **Cool-Down & Debrief** | 5 min | **Group Reflection - Seated Circle**  • Walking cool-down (2 min): pairs pass the ball while walking slowly around the court.  • Debrief questions (3 min):  – Why did the teacher ask you to start near different corners?  – What happened when two teammates stood in the same area?  – As a defender, which attacker were you responsible for - and how did you decide?  • Teacher closes: "Next lesson, we keep the same ideas - but we start introducing forward movement toward the goal." |

**Materials:** Soft handballs (1 per group of 6); cones (4 per court + 2 for each goal); bibs.

**Lesson 4 - Direct Instruction Group (Handball)**

**Operational Principles (Bayer, 1994)**

• ATK - Ball possession maintenance: passing to a moving target; immediate movement after passing

• DEF - Ball recovery: closing passing lanes through sliding defensive shape

• DEF - Goal protection: two-player defensive coverage and individual assignment

**Lesson Objectives**

• ATK: Execute the pass-and-move pattern - pass to a moving target and immediately relocate

• DEF: Execute defensive sliding with a partner - maintain shape, communicate roles, avoid crossing feet

| **Phase** | **Time** | **Description** |
| --- | --- | --- |
| **Warm-Up** | 8 min | **Pairs Passing Walk (Review)**  • Organization: pairs; 1 ball per pair; students walk side by side across the gymnasium.  • Task: continuous overarm throw between partners while walking - focus on grip (fingertips spread) and wrist snap.  • 2-word corrective cues only: "Snap wrist," "Step through," "Fingertips".  • Progression after 3 min: increase to jogging. |
| **Instruction & Modelling** | 7 min | **Explicit Teaching - Pass-and-Move + Defensive Sliding**  Pass-and-move (offensive):  • Teacher calls 2 volunteers. Demonstrates: Player A passes to B → A immediately sprints to a new spot → B passes back to A at the new spot.  • Demonstrated 3 times: walking speed → jog speed → with verbal cue "Pass - move!"  • Teacher explains why: "If you stand still after passing, your defender can ignore you. If you move, they must choose: follow you, or the ball?"  Defensive sliding (defensive):  • Teacher calls 2 more volunteers. Demonstrates stance: knees bent, feet shoulder-width, weight forward.  • Demonstrates sliding footwork: "Move the foot in the direction you want to go first, then bring the other foot. Never cross your feet."  • All students shadow-practice without a ball: 10 slides left → 10 slides right; 2 rounds. |
| **Guided Practice** | 22 min | **Two Concurrent Stations (11 min each; switch)**  Station 1 - Pass-and-Move Pairs:  • Organization: pairs; 1 ball; two movement cones placed 5m to the left and right of Player A's starting position.  • Task: A passes to B (6m apart) → A immediately sprints to one of the two cones → B passes back to A at the cone. 10 sequences, then swap roles.  • Teacher feedback: observe 3 consecutive sequences, then give one collective cue to the pair. Focus: does A move before B releases the return pass? Does B lead A with the pass (not behind)?  Station 2 - Defensive Sliding 2v2 (walking pace):  • Organization: 2 attackers walk side-by-side passing slowly; 2 defenders slide in front maintaining a compact line.  • Task: defenders must keep the central defender on the ball and the side defender on the second attacker. Verbal cue required: "I have ball!" / "I have player!"  • Attackers walk only - the emphasis is on defensive shape, not winning the ball.  • Teacher feedback: every 3 repetitions - check footwork (no crossed feet), check verbal communication, check side defender does not chase the ball. |
| **Collective Game** | 8 min | **4v4 + Goalkeeper - Regulation-style Small-Sided Game**  • Organization: court ~15m × 14m; 2 cone goals 2m wide; student goalkeeper rotates every 4 minutes or after suffering goals.  • Rules: standard handball rules simplified - overarm throw, ≤ 3 steps after catching, no contact.  • No freeze moments - teacher observes and notes for post-game brief feedback (30 sec). |
| **Cool-Down & Review** | 5 min | **Teacher-Led Recap**  • Walking cool-down (2 min): pairs pass the ball while walking slowly around the court.  • Review questions (teacher asks; students answer):  – After you pass the ball, what do you do immediately? [Expected: move to a new spot]  – When two defenders work together, who covers the ball and who covers the second attacker? [Expected: nearest to ball → ball; other → second player] |

**Materials:** 11 soft handballs; 16 cones (station boundaries + movement markers); 2 cone goals; bibs.

**Lesson 4 - Direct Instruction Group (Volleyball)**

**Volleyball Analogs Operational Principles**

• ATK - Ball maintenance: 3-touch sequence (reception→ setting → attack) to control the ball before attacking

• DEF - Court protection: defense with movement from the ready position

**Lesson Objectives**

• ATK: Execute and sequence the 3-touch chain (reception→ setting → attack); priority sequence completion over result

• DEF: Apply defense - correct positioning, communication of roles and responsibility for the area

| **Phase** | **Time** | **Description** |
| --- | --- | --- |
| **Warm-Up** | 8 min | **Self-Toss and Overhead Set Control**  • Organization: students spread out individually around the gym; 1 ball per student (or 1 per pair).  • Task: self-throw the ball upwards (approximately 3 m), let it drop lightly, pass the ball to yourself with an overhead pass; catch the ball after each contact. Advance with 3 consecutive passes without catching the ball.  • Teacher instruction reminder: "Fingers spread. Elbows pointing forward. Thumb of one hand close to the thumb of the other. Finish with elbows extended and hands flat. The ball should be above and in front of the head."  • 2-word corrective cues only: "Elbows extended", "Thumbs close", "Fingers spread". |
| **Instruction & Modelling** | 7 min | **Explicit Teaching - Offensive build-up from the overhead pass**  • Teacher demonstrates (with volunteer): (1) first touch: controlled reception; (2) second touch: overhead set to a teammate; (3) third touch: controlled send-over.  • Teacher names each touch clearly: "Touch 1: receive. Touch 2: set. Touch 3: send-over." The students repeat the names aloud.  • Key cue: "We are learning the sequence first - it doesn't matter if the ball falls."  Defense:  • Teacher on the opposing court with 2 volunteers: "This player is the defender. He stays in specific zones to defend the opponent's attack that are not blocked."  • The teacher demonstrates the defense scenario: the ball is launched into the opposing court → the defender, from the defense position, must defend it. High balls should be defended with an overhead pass, and lower balls with a forearm pass or a one-handed pass.  • Communication rule introduced: the defending player must shout "mine!" before playing the ball. |
| **Guided Practice** | 22 min | **Two Concurrent Stations (11 min each; switch)**  Station 1 - Trio Touches (without net):  • Organization: 3 players in a triangle (approximately 4 m between each); 1 ball per trio.  • Task: Player A throws gently overhand to B → B passes with an overhand touch to C → C lifts overhand to A → A catches. Rotate positions every 3 minutes.  • Teacher feedback protocol: stop a trio every 4–5 repetitions; give a collective cue.  Station 2 - Defense Formation Practice (low net at 1.8 m):  • Organization: 1 student on the defensive side; teacher or student throws the ball on the opposite side.  • Task: the thrower throws a ball lightly overhand. The defender must react to the ball as quickly as possible. Rotate the students after the defender performs 5 actions.  • Teacher feedback protocol: "React faster to the ball." "Defend the high ball so your team has time to reorganize the next play." |
| **Collective Game** | 8 min | **4v4 across Low Net (1.8m)**  • Organization: court ~9m × 9m court; low net; teams of 4.  • Rules: 3 touches are encouraged, but not mandatory; only serve from under the baseline; if the player taking the first touch needs to, they can hold the ball and continue the game normally; game with rules similar to the formal game.  • The teacher offers positive reinforcement during the game for successful 3-touch sequences and correct communication. |
| **Cool-Down & Review** | 5 min | **Teacher-Led Recap**  • Static stretching: forearms (wrist extension), shoulders (cross-body), calves.  • Review questions:  – What is the order of the 3 touches? [Expected: reception → setting → attack]  – In defense, why should we stay in the ready position? [Expected: to get to the ball in time for the save]  • Teacher closes: "In the next class, we will build on this sequence and focus on forearm passing." |

**Materials:** 18 soft volleyballs; low net at 1.8m; cones for court boundaries.
